# Demystifying non-invasive approaches for screening jaundice in low resource settings: a review

**DOI:** 10.3389/fped.2023.1292678

**Published:** 2023-11-20

**Authors:** Umme Abiha, Dip Sankar Banerjee, Saptarshi Mandal

**Affiliations:** ^1^ Department of Smart Healthcare, Indian Institute of Technology, Jodhpur, India; ^2^ All India Institute of Medical Science, Jodhpur, India; ^3^ Computer Science and Engineering, Indian Institute of Technology, Jodhpur, India; ^4^ Transfusion Medicine and Blood Bank, All India Institute of Medical Science, Jodhpur, India

**Keywords:** physiological jaundice, kernicterus, bilirubin, icterometry, visual inspection, transcutaneous bilirubinometers

## Abstract

All national and international pediatric guidelines universally prescribe meticulous bilirubin screening for neonates as a critical measure to mitigate the incidence of acute bilirubin encephalopathy (ABE) and Kernicterus. The prevailing gold standard for jaundice detection in neonates necessitates invasive blood collection, followed by subsequent biochemical testing. While the invasive procedure provides dependable bilirubin measurements and continues to be the sole gold standard diagnostic method for assessing bilirubin concentration. There exists a pressing need to innovate non-invasive screening tools that alleviate the sampling stress endured by newborns, mitigate iatrogenic anemia, and expedite the turnaround time for obtaining results. The exploration of non-invasive modalities for bilirubin measurements is gaining momentum, driven by the overarching goal of minimizing the number of pricks inflicted upon neonates, thereby rendering screening a swift, efficient, comfortable, and dependable process. This comprehensive review article delves extensively into the array of non-invasive approaches and digital solutions that have been proposed, implemented, and utilized for neonatal bilirubin screening, with a particular emphasis on their application in low-resource settings. Within this context, the review sheds light on the existing methodologies and their practical applications, with a specific focus on transcutaneous bilirubin meters. Moreover, it underscores the prevailing open challenges in this domain and outlines potential directions for future research endeavors. Notably, the review underscores the imperative need for robust educational programs targeted at both families and healthcare personnel to expedite the process of seeking timely care for neonatal jaundice. Additionally, it underscores the necessity for the development of enhanced screening and diagnostic tools that can offer greater accuracy in clinical practice.

## 1. Introduction

**Figure 1:**
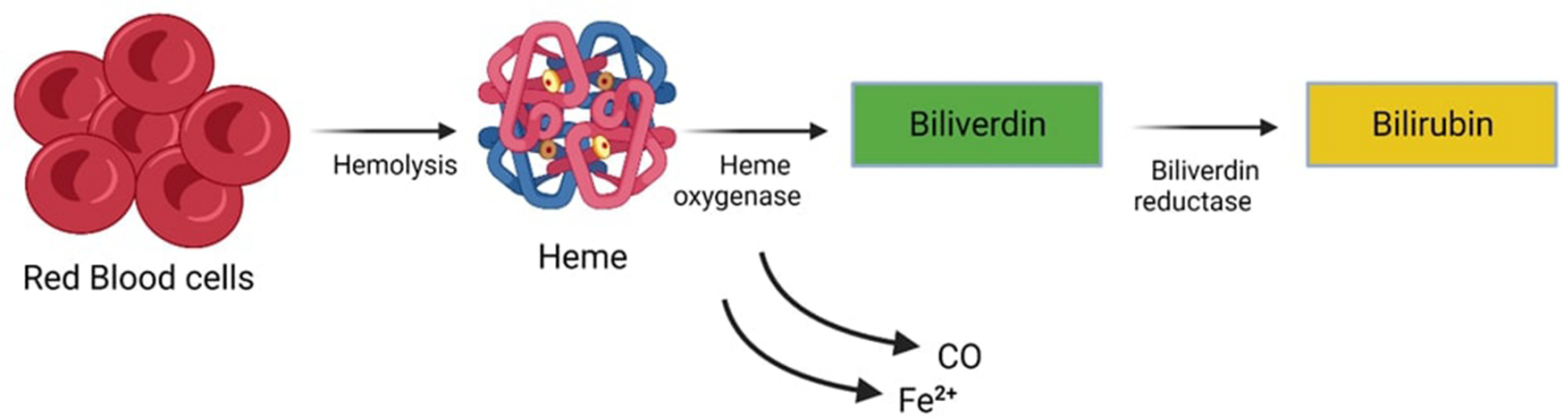
Metabolic imbalance characterized by bilirubin synthesis surpassing hepatic-enteric bilirubin clearance.

Newborns typically exhibit elevated bilirubin levels during their initial days of life, a condition known as Physiological Jaundice. However, this physiological jaundice can be exacerbated or prolonged due to various factors. Unconjugated (indirect) hyperbilirubinemia represents a prevalent and generally benign condition frequently observed in neonates. Jaundice (icterus neonatorum), manifests through noticeable effects on various bodily tissues. One of the prominent indicators is the impact it has on the skin, sclera (the white part of the eyes), and mucous membranes. This condition results from a metabolic imbalance where bilirubin synthesis surpasses hepatic-enteric bilirubin clearance, as illustrated in (Figure 1). The immaturity of the neonate's blood-brain barrier renders it permeable to a substantial bilirubin influx into the brain, thereby posing the potential risk of inducing a spectrum of irreversible cerebral injuries. These injuries may progress to acute bilirubin encephalopathy and culminate in kernicterus, a chronic form of bilirubin encephalopathy. Neonatal Jaundice impacts a significant portion of the neonatal cohort, affecting approximately 60% of full-term infants and 80% of preterm infants (1, 2). This prevalence translates to a significant number of infants, roughly 140 million born worldwide each year, developing Jaundice within the first two weeks of life (3). The threshold for clinically significant Jaundice, as measured by Total Serum Bilirubin (TSB) levels, varies based on postnatal age, race, comorbidity, and prematurity (4).

**Figure 2:**
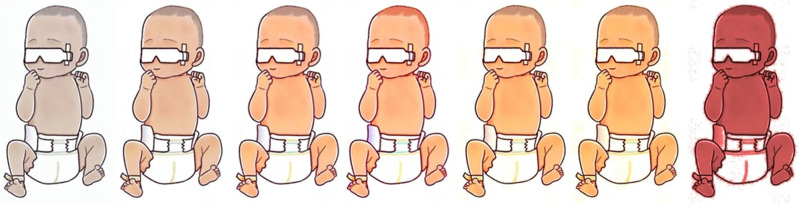
Progression from clinically significant jaundice to severe hyperbilirubinemia.

Contrary to common perception, bilirubin possesses valuable antioxidant properties. In vitro studies have demonstrated its ability to engage in an oxidation-reduction cycle with biliverdin, enabling it to remain active even at nanomolar concentrations. Furthermore, bilirubin exhibits robust superoxide and peroxyl radical scavenging capabilities. However, uncontrolled or rapid increases in bilirubin levels can reach neurotoxic levels with potentially fatal consequences. Hence, maintaining an equilibrium between the protective aspects of serum bilirubin and the risk of bilirubin-induced neurotoxicity is paramount for the well-being of jaundiced neonates. Cholestatic jaundice, particularly in its conjugated (direct) form, typically signifies underlying hepatic or biliary pathology. It is not uncommon for total serum bilirubin (TSB) to surpass age-specific high-risk thresholds, necessitating prolonged monitoring, potential rehospitalization, and a range of preventive measures (2, 3). Figure 2, elucidates the progression from the initial stages of clinically significant jaundice to the more advanced state of severe hyperbilirubinemia.

**Figure 3:**
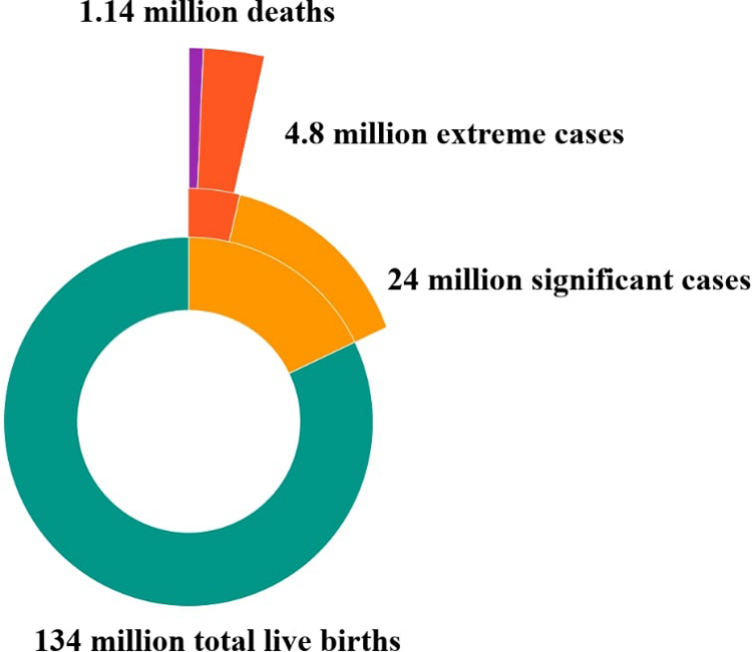
Global impact of neonatal jaundice [subsets were calculated based on the percentage data from Bhutani (2010) applied to the global denominator of 2022 using the mathematical modeling framework developed by Bhutani to estimate the global burden of severe jaundice] (2).

Mathematical modeling tools offer a means to estimate both fatal and non-fatal health outcomes associated with neonatal jaundice, providing valuable insights into its impact on mortality and morbidity. Bhutani et al. (Figure 3) were the first to assess the global burden of severe jaundice through this approach. Their model predicted that approximately 18% (or 24 million) of the 134 million live births in 2010 experienced clinically significant jaundice. Moreover, 0.481 million late-preterm and term neonates developed extreme hyperbilirubinemia (TSB *> *25 mg/dl), resulting in 0.114 million deaths and over 0.063 million survivors with moderate to chronic long-term neurological impairments (2).

It is worth noting that there is a notable absence of global and national neonatal population data, which are instrumental in informing policy decisions pertaining to neonatal care. Regrettably, no other records of global neonatal population studies have been identified at this time. Numerous non-invasive interventions have been proposed for bilirubin screening and diagnosis, particularly in resource-constrained settings. Among these, Transcutaneous bilirubinometers have garnered significant attention over the past three decades. Additionally, other non-invasive approaches, such as Visual inspection, Icterometry, Digital imagery, and Mobile phone applications, have been explored. This paper provides a comprehensive overview of available non-invasive technologies, with a particular focus on Transcutaneous bilirubin meters. It also addresses the current status of neonatal jaundice in LIC's and LMIC's, discussing economic, social, and technological barriers, as well as highlighting open research challenges in this field.

## 2. Administrating the status of neonatal jaundice in economically challenged low and lower-middle income countries

Numerous authoritative entities, such as the American Association of Pediatrics (AAP) and the UK National Institute for Health and Care Excellence (NICE), have proffered guidelines pertaining to the management of jaundice (1, 5–15). In high-income countries (HICs), the incidence of severe hyperbilirubinemia has markedly declined since the 1990s, owing to advancements in preventive measures and treatment options (16, 17). Current epidemiological evidence, derived from population-based studies and registries, places the estimated incidence of severe hyperbilirubinemia in HICs at 31.6 per 100,000 live births (95% CI: 11.8–51.3). Concomitantly, the incidence rates for Acute Bilirubin Encephalopathy (ABE) and Chronic Bilirubin Encephalopathy (CBE) range from 1.0 to 3.7 and 0.4 to 2.7 per 100,000 live births, respectively (18–22). In stark contrast, low- and middle-income countries (LMICs) grapple with distinct challenges, as they often lack uniform protocols for classifying and managing hyperbilirubinemia. This results in pronounced disparities in protocols across different regions, impeding comparative analysis. In LMICs, classification systems for hyperbilirubinemia are primarily developed, with the exception of Malaysia, which has adopted the NICE and AAP guidelines, incorporating international research findings and locally generated evidence (23, 24). It is unfortunate that documentation and record-keeping pertaining to Neonatal Jaundice (NNJ), ABE, and CBE incidence in LMICs are frequently inadequate and inconsistent (15–22, 25–26).

A recent modeling study conducted by the Child Health Epidemiology Reference Group (CHERG) undertook the estimation of neonatal mortality risk, including survival with kernicterus, on a global and regional scale. This estimation was predicated on country-specific and regional prevalence rates of Rh- positive infants born to Rh-negative mothers, G6PD deficiency, moderate-to-late preterm births, and infants lacking all three factors. The CHERG study posits that mortality rates due to Rh disease and/or extreme hyperbilirubinemia (EHB; TSB *> *25 mg/dl) are estimated to be 119 per 100,000 live births in Eastern Europe/Central Asia, Latin America, sub-Saharan Africa, and South Asia—figures significantly higher than the 1 per 100,000 live births reported in HICs (2). Likewise, the prevalence of kernicterus is substantially higher in the same four regions, with an estimated rate of 73 per 100,000 live births, compared to the 10 per 100,000 live births reported in HICs.

Diagnosing and managing jaundice in resource-constrained settings remain formidable due to multiple factors, including limited resources, dearth of trained personnel, and cultural and socioeconomic barriers. These circumstances engender suboptimal screening, misdiagnosis, and inappropriate treatment, thereby jeopardizing the well-being of neonates. Additionally, in certain cultural contexts, jaundice is not immediately recognized as a medical concern, and families may defer seeking medical attention until the condition deteriorates. Financial constraints may further impede access to healthcare services, resulting in delayed treatment. Issues also persist regarding the follow-up and monitoring of jaundiced neonates, with inadequate surveillance potentially leading to the oversight of severe jaundice cases necessitating urgent intervention. To address these challenges in low-resource settings, various interventions have been instituted, encompassing community-based screening initiatives, healthcare professional training and education, and enhanced access to diagnostic tools and treatment modalities. Long-standing efforts have been dedicated to heightening awareness and educating families on the imperative of seeking medical attention for jaundiced neonates.

The global prevalence of neonatal jaundice has spurred the development of comprehensive health programs aimed at its mitigation, both on a global scale and within India. Globally, the World Health Organization (WHO) has orchestrated several initiatives to combat neonatal jaundice, advocating for early detection and treatment via phototherapy and exchange transfusions. The organization has also devised guidelines to guide healthcare professionals in resource-limited nations, endorsing the use of cost-effective phototherapy devices and the training of personnel in diagnosis and treatment.

In India, neonatal jaundice presents a significant public health concern due to the country's high birthrate and underdeveloped healthcare infrastructure. The Indian government has implemented various programs to address this issue, including the National Rural Health Mission (NRHM), which strives to train healthcare providers and equip primary health centers with phototherapy devices. The Janani Shishu Suraksha Karyakram (JSSK) further extends care and treatment for jaundiced newborns free of cost. While these initiatives have measurably reduced the prevalence of neonatal jaundice, there persists a need for continued research and development, with an emphasis on devising more precise and cost-effective diagnostic tools for early detection and intervention. In summation, the collective efforts of global and Indian programs have yielded marked improvements in the health outcomes of neonates, yet ongoing endeavors are indispensable to alleviate the burden of this condition on newborns and their families. This assumes greater relevance in light of the United Nations' Sustainable Development Goals (SDGs), which encompass a comprehensive agenda for the survival, well-being, and enduring health of all neonates (27). The prompt identification of clinically significant hyperbilirubinemia is of paramount importance in averting kernicterus (28). Phototherapy remains the conventional approach to hyperbilirubinemia treatment, although exchange transfusions may be requisite in severe cases (29). While exchange transfusions are infrequent in high-income nations, it's risk persists in low-income and lower-middle-income countries (26). However, the early recognition of hyperbilirubinemia encounters hurdles in resource-scarce settings, characterized by prevalent home births and challenges associated with post-discharge follow-up (30). Poor healthcare-seeking behavior, parental unawareness, and logistical complexities can contribute to treatment delays, culminating in neonates presenting with extreme hyperbilirubinemia and moderate to severe stages of acute bilirubin encephalopathy, entailing elevated mortality and morbidity rates (31). In recent advancements, low-cost, minimally invasive instruments have surfaced for the detection of hyperbilirubinemia in regions with limited resources. Such instruments, encompassing smartphone-based technology and point-of-care methods for quantifying total serum bilirubin, show potential in the early identification of hyperbilirubinemia. Nevertheless, it is essential to emphasize the significance of educating healthcare practitioners and communities comprehensively to enhance their awareness of the principal risk factors associated with severe hyperbilirubinemia (32).

## 3. Morbidity and mortality factors

The elevated prevalence of neonatal hyperbilirubinemia in Low- and Middle-Income Countries (LMICs) can be primarily attributed to the relatively low rate of hospital-based childbirths within these regions. A significant portion of deliveries takes place beyond healthcare facilities, leading to the imperative for mothers and families to assume the role of recognizing jaundice in neonates (33). However, it is important to acknowledge that visual assessment alone proves inadequate for the accurate diagnosis of hyperbilirubinemia, particularly in terms of assessing its severity (34, 35). This challenge is further exacerbated in infants with pigmented skin, rendering the visual detection of jaundice even more intricate (36). Consequently, severe cases of neonatal jaundice predominantly manifest outside hospital settings due to the inherent difficulties associated with identifying jaundice at home (37). The delays in the commencement of treatment substantially contribute to heightened morbidity rates in LMICs (25), which is evidenced by the amplified occurrence of adverse clinical outcomes in infants residing in these areas.

Although adopting a proactive approach, such as the prompt administration of phototherapy, has proven effective in mitigating the severity of neonatal hyperbilirubinemia and ABE (38), it is important to recognize the existence of a threshold at which neonates progress to an irreversible condition known as the Kernicterus Spectrum Disorder (KSD). Neonates exhibiting a Bilirubin-Induced Neurologic Dysfunction (BIND) score below 4 are considered to have a reversible condition, and some infants with a BIND score ranging from 4 to 6 may still exhibit potential reversibility with appropriate treatment (39). As underscored by Olusanya et al. (25), various factors contribute to delays in seeking care, including a lack of recognition, knowledge, and access to suitable treatment among both caregivers and healthcare providers, financial constraints, and geographical distance. Regrettably, in many instances, care is only sought when the child begins to display signs of ABE (25).

**Figure 4:**
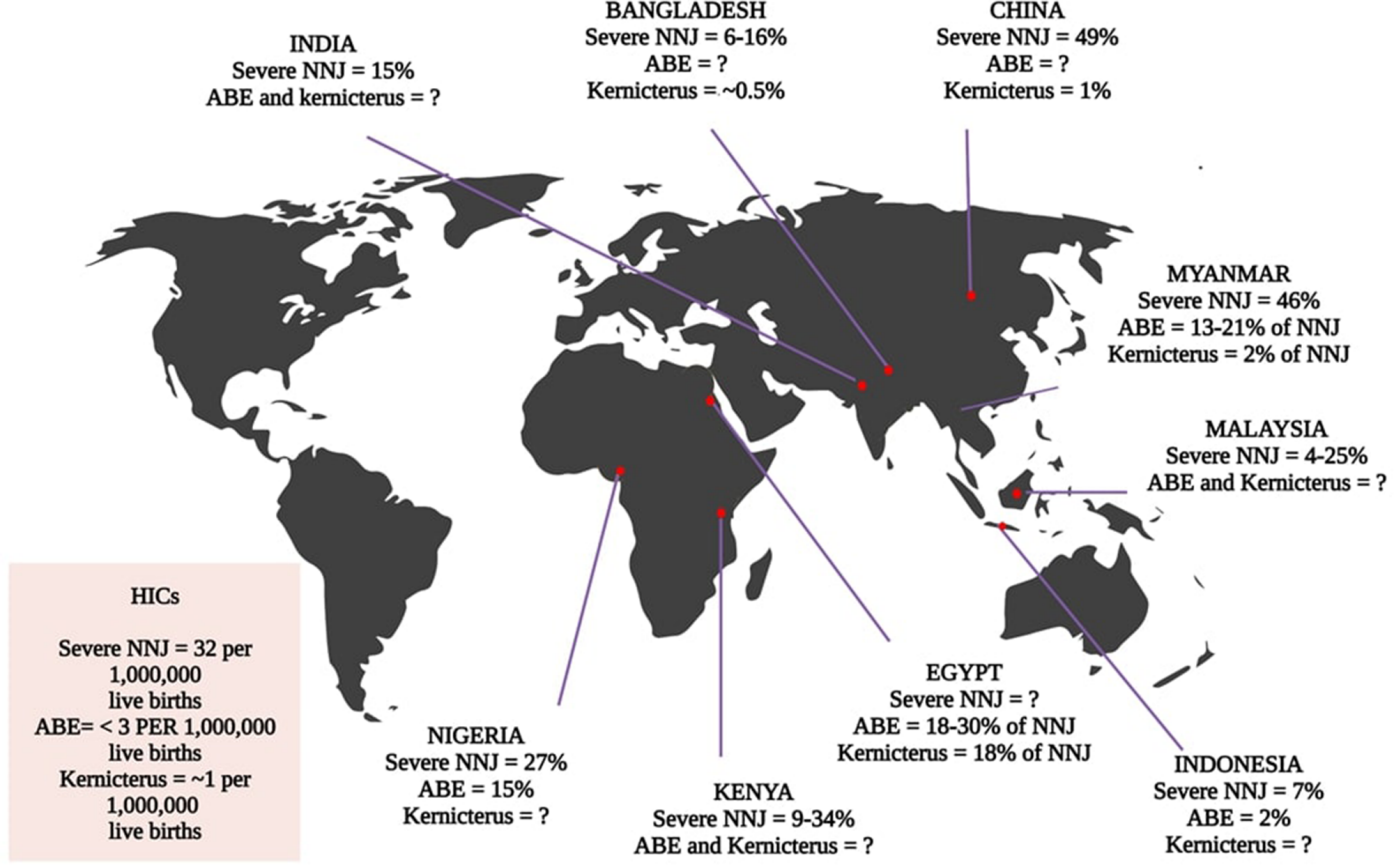
Incidence of severe neonatal jaundice (NNJ) in LMICs. The data is based on hospital statistics, as no national records were identified in the literature (24).

Healthcare providers sometimes recommend suboptimal or ineffective treatments, such as exposure to unfiltered sunlight, herbal remedies, glucose, and antibiotics (25, 39). Prenatal screenings may not consistently identify potential blood group incompatibilities or establish a plan to address Rhesus isoimmunization, and even when such screenings do occur, effective prophylaxis may not be readily accessible or affordable (40). Another form of delay elucidated by Olusanya et al. pertains to the absence of comprehensive, jaundice-specific care at healthcare facilities, which stems from the lack of essential guidelines, diagnostic tests, screening protocols for conditions like G6PD deficiency, and the capacity to monitor bilirubin levels effectively (25). As previously mentioned, all these factors are frequently associated with ineffective phototherapy (25, 41–45). Furthermore, infections, particularly umbilical sepsis arising from improper cord severing, inappropriate handling of neonates with non-sterile materials, and delivery in unhygienic environments, are commonplace in non-hospital settings (15). These factors heighten the risk of severe hyperbilirubinemia. Suboptimal treatment regimens result in wasted time and resources and an increased rate of exchange transfusions, along with the associated risks, in LMICs. The improper installation and maintenance of phototherapy devices further contribute to the subpar quality of treatment (43). A study conducted in Nigeria demonstrated that simple adjustments in the distance between the light source and the infant's skin, coupled with regular maintenance of phototherapy devices, could markedly elevate average irradiance levels from below the minimum requirement for conventional phototherapy (*<*10 µW/cm^2^/nm) to levels approaching those of intensive phototherapy (27.3 µW/cm^2^/nm) (43). Refer to Figure 4 for Severe NNJ in LMICs.

Research concerning the long-term follow-up of children affected by KSD in LMICs has revealed that surviving children contend with various challenges, including choreo-athetoid cerebral palsy, deafness, and auditory processing disorders (46–49). Nevertheless, conducting sustained follow-up assessments presents significant logistical challenges, and the available resources to address their ongoing needs remain limited (50, 51). For recent studies elucidating the serious outcomes associated with severe hyperbilirubinemia in selected African and Asian countries, please refer to Table 1.

**Table 1:** Neonatal jaundice in African and Asian countries.

Country	Severe NNJ incidence, %	ABE incidence, %	CBE and Kernicterus incidence, % of NNJ	NNJ deaths of newborns admitted, %	NNJ deaths with respect to all deaths, %	CFR due to NNJ, %	CFR due to ABE, %	Mortality rate	References
Egypt	NA	18% of NNJ 30% of NNJ	NA 18.1	NA	NA	10.5 6.5	59.1 22.4	NA	(52, 53)
Kenya	34.4 9.2	NA	NA	7.8 1.3	NA 5.7	22.7 14.3	NA	NA	(54, 55)
Nigeria	26.9	14.9	NA	3.5	NA	13.0	23.5	NA	
China	49.1	NA	0.9	NA	NA	NA	NA	NA	(56)
Bangladesh	15.7 5.9	NA	0/5 NA	0.6 0.2	NA 1.1	3.8 3.9	55.6 (of CBE) NA	NA	(57, 58)
India	15.3 NA	NA	NA	1.0 NA	4.4% NA	6.7 NA	NA	NA 730/100,000 Live births	(59, 60)
Myanmar	46.0 NA	NA 12.7% NNJ in A 21.2% NNJ in B	NA 2.0% NNJ in A 1.5% NNJ in B	NA	NA	NA 7.2 (A) 11.2 (B)	NA 46.9 (A) 25.0 (B)	NA	(61, 62)*
Malaysia	25–30 3.8	NA	NA	NA	NA	NA	NA	NA	(24)
Indonesia	6.8	2.2	NA	1.6	NA	24.2	74.9	NA	

NA, Not available.

ABE is a clinical syndrome characterized by symptoms such as lethargy, hypotonia, and poor sucking, which may progress to hypertonia with opisthotonos and retrocollis, accompanied by a high-pitched cry and fever, and potentially leading to seizures and coma. CBE and kernicterus represent the clinical consequences of ABE, encompassing irreversible brain damage manifested as athetoid cerebral palsy, sometimes accompanied by seizures, developmental delay, hearing impairment, oculomotor disturbances, dental dysplasia, and intellectual disability. Histologically, CBE is distinguished by the deep-yellow staining of neurons and neuronal necrosis within the basal ganglia and brainstem nuclei. The investigation conducted by Arnolda et al. (62) * was carried out in two distinct hospitals, identified as Hospital A and Hospital B.

## 4. Non-invasive approaches for jaundice screening and their limitations

**Figure 5:**
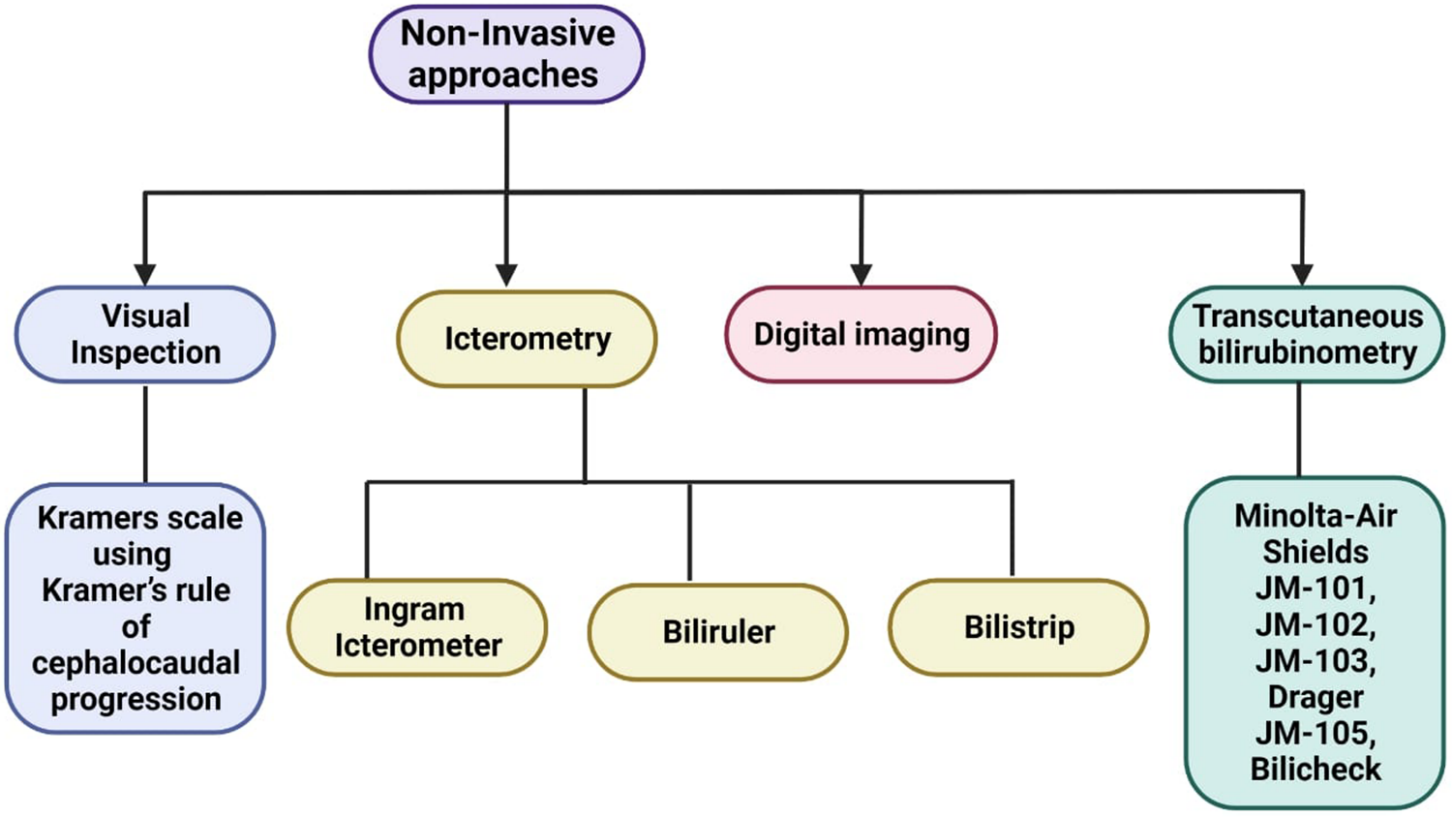
Non-invasive approaches for screening and diagnosing neonatal jaundice.

This section presents a review on the existing non-invasive modalities and methodologies employed in the screening of neonatal jaundice, as well as the estimation of bilirubin levels in neonates along with the its limitations. The primary themes of interest within this section are outlined in Figure 5.

### 4.1. Visual inspection

In neonatology, the visual inspection serves as a preliminary screening tool for the assessment of jaundice in newborns. Neonatal jaundice is characterized by the accumulation of bilirubin, that manifests as a yellowish discoloration of the skin and the sclera of the eyes. The initial step in diagnosing jaundice involves a comprehensive physical examination of the neonate. Visual inspection constitutes a facet of this evaluation, wherein healthcare practitioners gauge the severity of jaundice by discerning the extent of yellowish pigmentation in the neonate's skin and ocular tissues, thereby facilitating the determination of the jaundice's severity (63).

**Figure 6:**
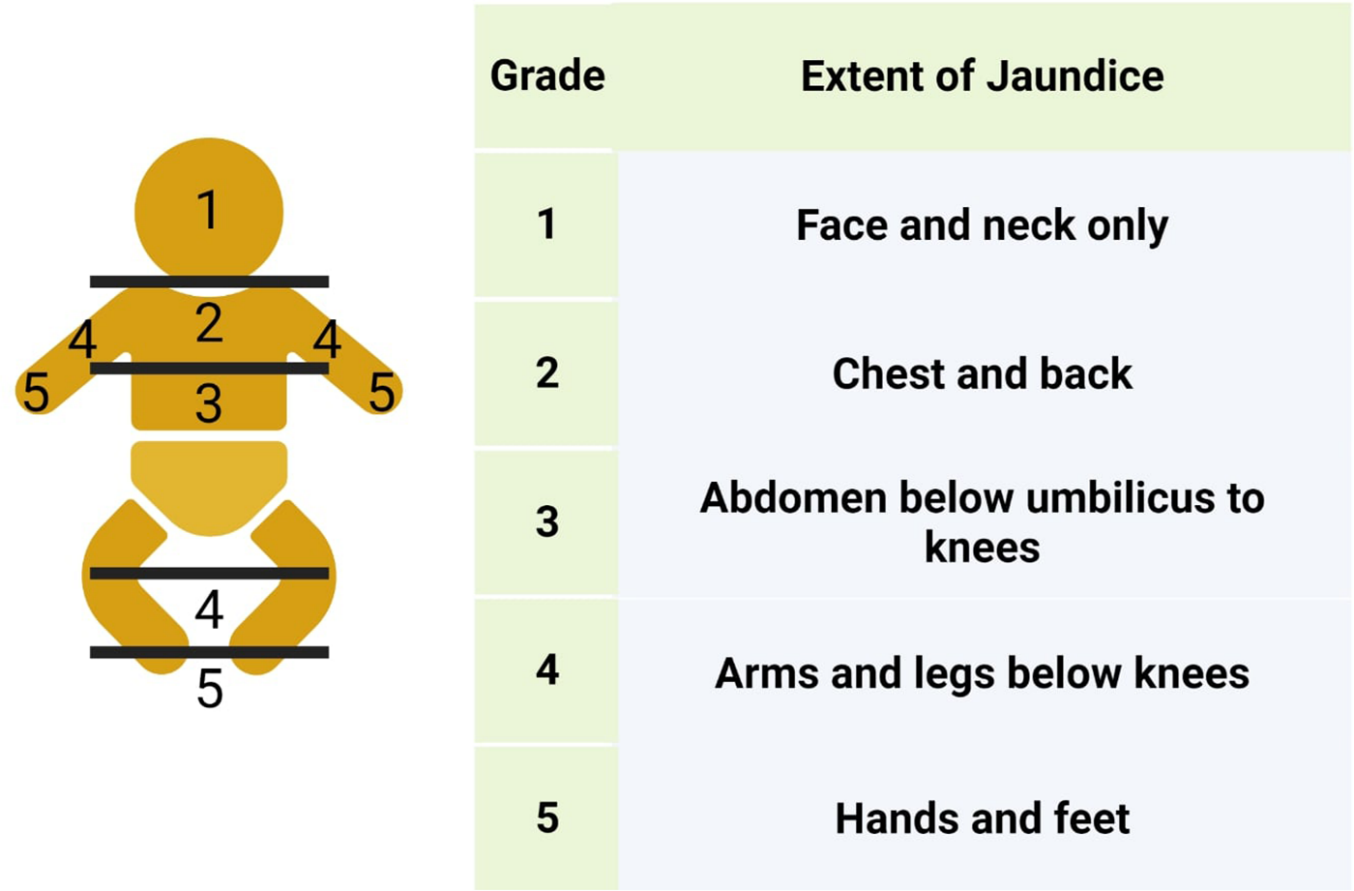
Kramer’s rule of cephalocaudal progression.

This assessment methodology, known as the Kramer scale, encompasses the comparison of jaundice intensity on the neonate's skin, guided by Kramer's principle of cephalocaudal progression. The Kramer hierarchy postulates that the progression of yellow discoloration transpires in a cephalocaudal fashion, encompassing five dermal zones: Head and neck, upper trunk, lower trunk and thighs, arms, and lower legs, and palms and soles, as illustrated in Figure 6.

Clinical observations have indicated that jaundice in neonates initially manifests on the facial region. As the total serum bilirubin (TSB) level escalates, this manifestation extends to encompass the chest, abdomen, and extremities. Empirical validation of this observation has been achieved through transcutaneous bilirubin measurements. Knudsen postulated that the cephalocaudal progression of jaundice arises from conformational alterations within bilirubin-albumin complexes. Although the initial binding of bilirubin to albumin occurs rapidly, the ultimate conformational changes may require up to eight minutes (64). Consequently, bilirubin with a weaker affinity to albumin may circulate in the bloodstream, preferentially relocating to proximal body parts compared to the subsequent involvement of distal regions. Bilirubin with weaker albumin binding has a higher likelihood of precipitating as bilirubin acid within phospholipid membranes present in the skin and subcutaneous tissues, elucidating the early appearance of jaundice on the face as opposed to the abdomen or lower extremities (64). This conceptual framework has been employed in clinical practice to assess the severity of jaundice. Purcell and Beeby conducted an investigation to examine, if the cephalocaudal progression of jaundice in neonates was associated with variations in skin temperature and skin perfusion across five anatomical sites: the forehead, sternum, lower abdomen, mid thigh, and sole. It was observed that newborn infants exhibit a preference for increased blood flow to their head and the proximal regions of their body during the initial days of life. This phenomenon results in elevated temperatures and an enhanced deposition of bilirubin at these specific sites (65). However, it is essential to acknowledge the limitations of Kramer's rule and Visual Inspection in order to better understand its applicability and potential shortcomings in clinical practice.

*Limitations:*

1. Inter-individual Variation: One of the primary limitations of Kramers rule of cephalocaudal progression is the significant inter-individual variation seen in neonates. While the guideline suggests that jaundice typically appears first in the face and then progresses downwards, this progression is not consistent in all newborns. Some infants may exhibit jaundice in areas other than the face initially or may follow a different pattern of progression. This variability can lead to diagnostic confusion and potentially delayed intervention in some cases (66).
2. Ethnic and Racial Differences: Kramers rule of cephalocaudal progression is primarily based on observations in populations of European descent. It may not be equally applicable to neonates of different ethnic or racial backgrounds who may exhibit variations in the timing and pattern of jaundice presentation. Failure to account for these differences can result in misinterpretation of clinical signs and suboptimal management strategies (9). In a cross-sectional study of 315 neonates under 28 days of age of Black descent, conducted by Dionis et al., it was found that Kramer's method demonstrates a noteworthy positive predictive value. Nevertheless, its overall predictive capability cannot be considered substantial due to its low sensitivity and negative predictive value. Consequently, the clinical assessment based on Kramer's method should not be recommended for neonatal jaundice screening in this population. Further investigations are warranted to explore the efficacy of alternative non-invasive techniques in the detection of neonatal jaundice among this population (67).
3. Limited Sensitivity: The rule's reliance on visual assessment alone to determine the cephalocaudal progression of jaundice may lack the sensitivity required to identify subtle or early signs of hyperbilirubinemia. In some cases, laboratory tests such as serum bilirubin levels or transcutaneous bilirubinometry may be necessary to accurately assess the severity of jaundice, especially in infants with darker skin pigmentation where clinical examination may be less reliable (68).
4. Complex Etiology, Age and Developmental factors: Neonatal jaundice can have various underlying causes, including physiological, pathological, and genetic factors. Kramers rule does not account for the etiological diversity of neonatal jaundice, which can have implications for treatment decisions. Relying solely on cephalocaudal progression assessment may overlook underlying pathological conditions that require specific interventions (69). Moreover, the progression of jaundice can also be influenced by factors such as the age and developmental stage of the neonate. Premature infants, for example, may exhibit different patterns of jaundice compared to full-term infants (70).
5. Risk of Overlooking Severe Jaundice: In some instances, neonates may develop severe hyperbilirubinemia without obvious clinical signs of jaundice in the face. Focusing solely on the cephalocaudal progression may lead to delayed diagnosis and treatment of severe jaundice, which can have serious neurological consequences, such as kernicterus. Sampurna et al., conducted an investigation aimed at assessing the potential utility of Kramer's score in the identification of infants requiring phototherapy. Their findings led to the conclusion that the Kramer score may not serve as a valid method for distinguishing between infants necessitating phototherapy and those for whom this treatment may not be warranted (71).

As a preliminary screening tool, it necessitates the consideration of several inherent limitations when interpreting its outcomes. Heavy reliance on the subjective judgment of healthcare providers introduces variability in individual perceptions, thereby giving rise to inconsistent diagnoses (72). Although methodologies such as the Kramer scale may serve as initial screening aids, blood tests constitute the confirmatory step for diagnosis and treatment planning. Moreover, the diagnosis of jaundice through visual inspection necessitates a TSB concentration surpassing 5–6 mg/dl (85–100 µmol/L) (73). However, it is worth highlighting that even seasoned neonatologists may occasionally misidentify infants with elevated TSB concentrations. According to the current multidisciplinary Dutch national guideline for neonatal jaundice identification and treatment, visual inspection serves as the primary screening tool for neonates receiving home-based care (74). In cases where the suspicion of severe hyperbilirubinemia arises from visual inspection, community midwives may opt to assess bilirubin levels in the neonate's blood, either collected by the midwife or through a specialized laboratory home service. Subsequently, treatment decisions for hyperbilirubinemia are determined based on the laboratory-based bilirubin (LBB) level, utilizing the nomogram outlined in the Dutch national guideline, which is adapted from the 2004 guideline of the American Academy of Pediatrics. Numerous studies have underscored the unreliability of visual inspection as a screening tool for neonatal hyperbilirubinemia (73–76). A substantial proportion of neonates, admitted from home with severe hyperbilirubinemia or acute bilirubin encephalopathy, experienced instances where neonatal jaundice either remained unnoticed or was misclassified by maternity care assistants, midwives, and/or parents (73, 75, 76). Hence, it exhibits reduced sensitivity, particularly in the detection of mild jaundice rendering it less sensitive in comparison to laboratory-based techniques.

Additionally, the method's accuracy diminishes notably when applied to individuals with dark skin, owing to the inherent challenge of discerning skin yellowing (9, 67). Consequently, this limitation may result in an underestimation of jaundice severity. It is noteworthy that ambient lighting conditions can exert an influence on the accuracy of visual inspection, potentially causing fluctuations in the perceived severity of jaundice (77). Furthermore, the method's inability to provide quantitative data on bilirubin levels precludes its utility in trend monitoring and the evaluation of treatment effectiveness. It is imperative to note that visual inspection alone lacks precision, which necessitates the integration of complementary diagnostic methodologies, such as laboratory-based assays which quantify the concentration of bilirubin, enabling healthcare practitioners to ascertain the severity of jaundice and determine the most suitable course of action. Table 2 (Studies on Non-Invasive Approaches for Neonatal Jaundice Estimation) provides a compilation of pivotal comprehensive investigations on various non-invasive approaches employed in the estimation of jaundice severity in neonates.

**Table 2:** Comprehensive studies on various Non-invasive approaches for estimating jaundice in neonates.

Non-invasive approaches	Number of studies	Sample size	Observation	References
Visual Inspection	12	3,077	Interobserver agreement among different levels of healthcare professionals, namely physicians, nurses, and parents, is notably low.	(34, 75, 78–87)
Ingram Icterometer (including Biliruler and Bilistrip)	5	3,656	In the past, icterometers were manufactured using different color batches, which might not have been standardized. More recent devices utilize color processing technology and have the potential to be valuable in clinical settings.	(88–92)
Transcutaneous Bilirubinometers	42	12,006	A reasonable correlation was observed between total bilirubin (TB) and TcB values when using different devices. However, older icterometers were manufactured from various color batches, potentially lacking standardization.	(88, 93–134)
Digital imaging or mobile applications	5	1,020	Correlation coefficients varied widely between mobile phone devices measurements and TcB. The digital technology is still in its development phase and not commercially available.	(135–139)

### 4.2. Icterometry

Icterometry represents a non-invasive technique employed to indirectly evaluate the degree of jaundice by visually assessing transcutaneous bilirubin concentrations within subcutaneous tissue and adipose layers.

#### 4.2.1. Ingram icterometer or gosset icterometer

The Ingram Icterometer, also known as the Gosset Icterometer, serves as a non-intrusive instrument for quantifying bilirubin levels. It consists of transparent plastic divided into five horizontal strips, each adorned with graded yellow lines (140). Employed since 1925 to gauge the severity of jaundice, this tool involves placing it against the patient's nose, wherein the blanched skin's color is juxtaposed with the graded yellow lines to ascertain the bilirubin concentration (141).

#### 4.2.2. Bili-ruler

**Figure 7:**
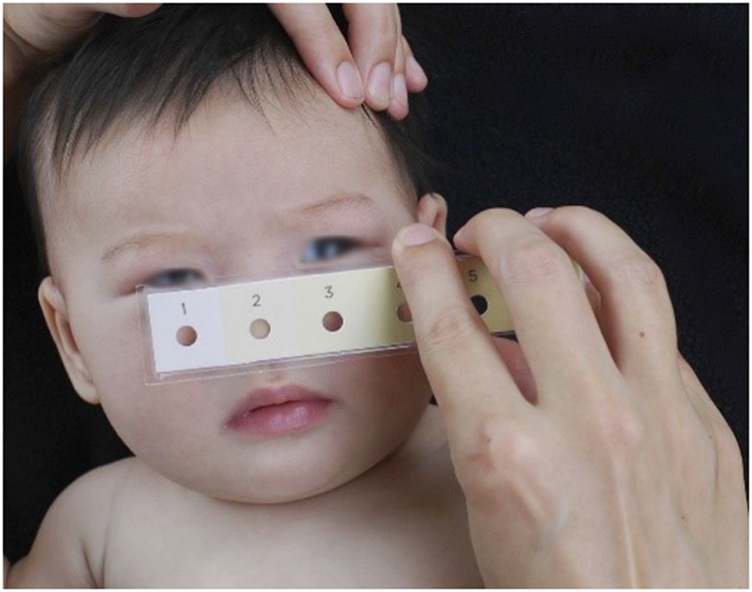
Utilization of bili-ruler to check neonatal jaundice (142).

The Bili-ruler represents an economical and reusable non-invasive apparatus, which can be sanitized between successive patient usages. It effectively enhances referrals from peripheral healthcare facilities to more advanced centers equipped with bilirubin testing and/or phototherapy capabilities. The Bili-ruler rectifies the limitations inherent in the original Gosset icterometer through the application of advanced digital color processing techniques and a human-centered design approach. This tool comprises digitally standardized and calibrated archival-quality paper strips, sequentially numbered from 1 to 6, progressively transitioning in hue towards yellow (refer to Figure 7). Notably, research has documented the development of an innovative Bili-ruler prototype, which was in-house engineered utilizing a semiflexible acrylic base and adherent acrylic film for affixing the color strips (142).

The color strips were generated through the acquisition of images depicting blanched skin from infants possessing known serum bilirubin levels, employing an X-rite ColorChecker Passport (142). This methodology facilitated the development of a digital camera calibration profile and standardized color output for subsequent processing. Subsequently, a comprehensive digital photograph repository was compiled, featuring infants exhibiting a spectrum of hyperbilirubinemia levels, spanning from none to severe. The highest score obtained from the Gosset icterometer was selected, employing a stepwise gradient to construct the color scale. The digital workflow and image processing operations were conducted using Adobe Photoshop and Lightroom applications, executed within the sRGB 16-bit color space. It is noteworthy that one of the prominent challenges associated with the original icterometer was the complexity of making decisions pertaining to color matching (143). To address this issue, the group devised a novel approach that replaced the linear color strips with a transparent circular window enveloped by a uniform color swatch. This redesigned configuration necessitated users to make simplified binary determinations (i.e., yes or no) concerning color matching. A similar design principle has previously been applied in Hemochek, an apparatus utilized for screening anemia based on the World Health Organization's Hemoglobin Color Scale, aimed at facilitating color matching (142). To employ the Bili-ruler, users are required to apply firm pressure to induce blanching of the infant's skin, subsequently assessing the hue of the underlying subcutaneous tissue. This process is iterated for each color segment on the ruler, ranging from 1 to 6, with users selecting the Bili-ruler score that most closely corresponds to the underlying skin color. Importantly, these measurements should be taken in natural light, preferably near a window, and devoid of fluorescent lighting.

#### 4.2.3. Bilistrip

**Figure 8:**
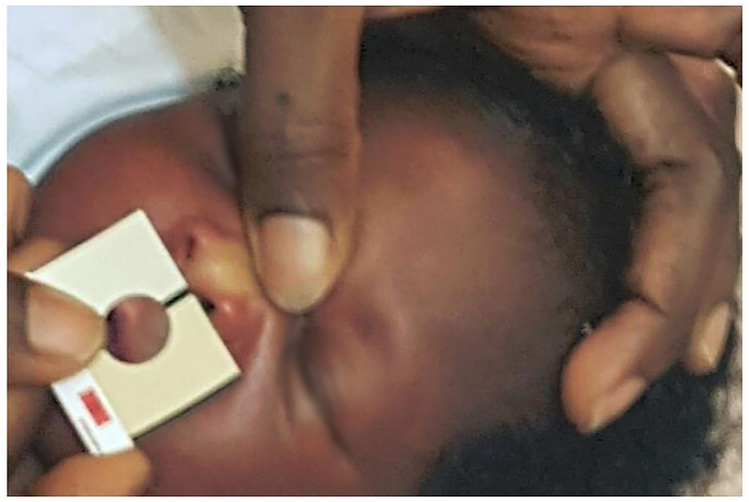
Utilizing bilistrip™ to screen jaundice (90).

The Bilistrip device emerges as an innovative two-color icterometer, presenting a promising instrument designed to enable mothers to discern neonates exhibiting clinically significant jaundice, thereby indicating the need for vigilant monitoring or treatment, as well as neonates who do not require immediate intervention for jaundice during their first week of life (see Figure 8). Neonates selected as color B by their mothers are deemed to be at heightened risk of hyperbilirubinemia, warranting priority for subsequent bilirubin assessment and potential treatment. Conversely, neonates designated as color A by their mothers are less likely to manifest clinically significant jaundice, barring instances where hemolysis is suspected. Numerous studies have explored maternal capacity to detect jaundice in their infants, primarily by observing yellowish skin and scleral discoloration (144–148). Significantly, maternal concern, often voiced by individuals lacking expertise in jaundice assessment, can be prompted by early indications of Acute Bilirubin Encephalopathy (ABE), such as feeding difficulties, infant irritability, and restlessness (25). It is crucial to acknowledge that, even in high-income countries, electronic TcB (Transcutaneous Bilirubin) devices intended for domestic utilization, though potentially more reliable, are unlikely to be offered to mothers due to their considerably higher cost, with a typical TcB device costing around $3,000 per unit, excluding operational expenses, compared to $17 for the Ingram icterometer (80, 134) or approximately $0.10 for a Bilistrip™ unit.

In a particular investigation concentrating on primarily Caucasian neonates after their discharge, an icterometer threshold of 2.5 demonstrated a sensitivity of 73%, specificity of 65%, a Positive Predictive Value (PPV) of 44%, and a Negative Predictive Value (NPV) of 87% in the identification of neonates with Total Serum Bilirubin (TSB) levels surpassing 12 mg/dl. In the context of TSB levels surpassing 17 mg/dl, the aforementioned study documented a sensitivity of 100%, specificity of 58%, a PPV of 12%, and a NPV of 100% (134). Conversely, a population-based investigation revealed that Bilistrip™ displayed a sensitivity of 91%, specificity of 24%, a PPV of 32%, and a NPV of 88% in the prediction of TSB levels surpassing 12 mg/dl. When TSB levels exceeded 17 mg/dl, Bilistrip™ exhibited a sensitivity of 100%, specificity of 21%, a PPV of 7%, and a NPV of 100%. This suggests that race does not significantly confound the utilization of icterometers in neonates during the first week of life, consistent with findings from other investigations (89, 134, 149–151).

Furthermore, a number of research studies have employed a 3-cut-off methodology with Ingram icterometers for the identification of neonates displaying significant jaundice (89, 134, 151, 152). In the majority of cases, the mean TSB corresponding to this threshold value approximated 10 mg/dl, which is consistent with the mean TcB of 10 mg/dl and the mean TSB of 11 mg/dl for color B. This concurs with the proposition that existing multi-shade icterometers can be effectively replaced by a simpler two-color icterometer like the Bilistrip. Some studies have assigned specific bilirubin levels to distinct shades of yellow on the icterometer (153). Nonetheless, it is important to emphasize that this practice is not indicative of a diagnostic approach at large.

*Limitations:*

Dr. Gosset openly acknowledged the limitations of the icterometer concerning its accuracy and its inability to distinguish between different types of jaundice. He emphasized the necessity of conducting blood sampling in cases of neonatal jaundice with a rapid onset, specifically within thirty-six hours of birth. The icterometer could not be employed for infants with nasal bruising, where the use of the gums was recommended instead. For children of non-Caucasian ethnicity, the icterometer could only indicate whether the jaundice was progressively worsening or not on a day-to-day basis, without providing precise measurements, and uncalibrated readings necessitated the use of blood samples (151, 154). (It is noteworthy that subsequent research published in India in 1991 contradicted this assertion.) A study involving 1,161 newborns with a gestational age of 35 weeks or more was conducted, and TSB levels were measured based on clinical indications. A novel JCard was used to take measurements made by both parents and pediatricians and finally compared with the TSB measurements. It demonstrated the ability to classify various bilirubin levels, but its accuracy was observed to decrease when dealing with high bilirubin levels. Notably, the diagnostic performance of parents using the JCard was found to be slightly less precise compared to that of pediatricians (155).

### 4.3. Transcutaneous bilirubinometers

Traditional visual inspection for neonatal jaundice is known to exhibit poor correlation with Total Serum Bilirubin (TSB) levels, particularly among non-Caucasian infants. Laboratory-based TSB testing, while considered the gold standard, presents challenges in resource-limited settings due to cost and a dearth of trained personnel. In response to these challenges, non-invasive transcutaneous bilirubin (TcB) devices have emerged as promising screening alternatives to visual inspection. TcB devices are validated, non- invasive screening tools designed to estimate serum bilirubin concentrations in neonates (156, 157). These devices employ visible light technology to assess bilirubin levels in the skin, providing a near estimate of the bilirubin concentration in the blood. Typically positioned on the infant's skin, most commonly on the forehead or sternum, TcB devices yield rapid readings in seconds. Their reliability and accuracy in predicting elevated bilirubin levels in neonates are well-established, and they offer advantages over traditional screening methods, such as visual inspection and icterometers. Moreover, they also obviate the need for subsequent blood draws solely required for screening and facilitating bedside use, thus enhancing convenience and reducing infant discomfort (158).

The process of obtaining a measurement of TcB is relatively straightforward, although the underlying physics and biology are intricate. TcB devices exhibit significant variations in design, but they all rely on the analysis of skin remittance, specifically diffuse reflectance spectra. When exposed to light of varying wavelengths emitted by the device, the skin interacts with this light, and the device possesses the capability to analyze the light that returns after it has undergone “processing” within the skin and subcutaneous tissue (159). The spectra of the reflected light depend on the concentration of various cutaneous chromophores, including melanin, collagen, oxygenated and reduced hemoglobin, and, notably, bilirubin (160). Technically, a “chromophore” refers to the part of a molecule responsible for its color due to its absorption and reflection of specific wavelengths, and the term is often used to refer to the entire molecule. The distinct absorption spectra resulting from various chromophores enable the calculation of their concentration through the analysis of reflected light, using a device-specific algorithm.

For the purpose of optical measurements, human skin can be conceptually treated as a layered structure comprising the epidermis (with a thickness of approximately 0.1 mm), dermis (around 0.5 mm thick), and subcutaneous tissue. Short wavelengths in the optical spectrum, typically falling within the 400–600 nm range, are primarily absorbed by specific chromophores, such as hemoglobin, melanin, bilirubin, and other pigments (161). The measurement device's ability to reflect and collect these wavelengths is crucial for accurately determining the baseline absorption of various pigments, facilitating the isolation of the specific chromophore of interest—namely, bilirubin. Bilirubin exhibits an absorption peak in the range of 450–500 nm, while melanin's absorption gradually diminishes as wavelengths increase from 400 nm to 840 nm (160, 162–166). Because epidermal melanin is a thin surface layer on the tissue, it acts as a light attenuation filter for both incoming and outgoing light, a factor that can be accounted for in the internal algorithm. Early research on the connection between serum bilirubin and cutaneous bilirubin, assessed through spectral analysis, was conducted by Ballowitz and Avery (167). Subsequently, Hanneman et al. at the Mechanical Engineering Department of Purdue University advanced the instrumentation for acquiring spectral reflectance data from the skin of human newborns (168, 169). Their approach involved the analysis of five distinct wavelengths, resulting in a noteworthy correlation coefficient of 0.93 between their method and total serum bilirubin (TSB) levels. Yamanouchi et al., in collaboration with Minolta Camera Company Ltd, introduced a prototype device featuring a digital processor that illuminated the skin and gauged color intensity (170). This device generated a numerical index, which exhibited a correlation with TSB. However, healthcare providers were required to translate this numerical index into a serum bilirubin equivalent through the utilization of a graphical chart or a conversion equation (171). Subsequent adaptations of this technology were introduced and commercialized under the names JM-101TM and JM-102TM. Analogous to the prototype, these devices generated an index value necessitating conversion into an estimate of TSB levels.

Several factors can influence the accuracy of TcB measurements, including skin color, gestational age, and the timing of assessment. Darker skin pigmentation can result in lower TcB readings, while premature infants may exhibit higher TcB values due to their thinner skin density. Additionally, the precision of TcB measurements may diminish after the initial 24 h of life, underscoring the importance of close bilirubin level monitoring during the early postnatal period (172). It is essential to emphasize that TcB measurements do not serve as a substitute for laboratory-based serum bilirubin level assessment, which remains the gold standard for diagnosing and monitoring neonatal jaundice (173). In situations where TcB measurements yield elevated results or clinical symptoms suggest significant jaundice, it is imperative to confirm the diagnosis and guide management through laboratory serum bilirubin level measurements. Several studies have explored the comparability of TcB measurements obtained from the forehead and sternum (110, 174–176). However, the Guideline Development Group (GDG) convened by the National Collaborating Centre for Women's and Children's Health (NCC-WCH) has recommended that measurements taken over the sternum are preferable for both parents and infants. This choice is motivated by the avoidance of potential challenges in obtaining readings from the forehead, such as wrinkling due to infant crying and the risk of ocular injury if the infant resists measurement on the forehead. Furthermore, it should be noted that discrepancies between TcB and TSB measurements become more pronounced when bilirubin levels exceed 250 µmol/L. Consequently, it is advised not to rely on TcB measurements for bilirubin levels exceeding 250 µmol/L; instead, serum bilirubin level measurements should be conducted to ensure accuracy (98).

The GDG has recommended against the use of transcutaneous bilirubinometers in preterm neonates (gestational age *< *35 weeks) due to their heightened vulnerability to kernicterus at relatively low bilirubin levels, coupled with the suboptimal performance of transcutaneous bilirubinometers in this population (177). Moreover, the accuracy of transcutaneous bilirubinometers in this subgroup remains unclear. To address this, the GDG advocates for the exploration of the BiliCheck and JM-103 devices specifically in jaundiced preterm neonates. While visual skin inspection by parents or clinical staff effectively rules out jaundice, it remains unreliable for assessing the depth of jaundice. However, the GDG acknowledges that transcutaneous bilirubinometers offer a non-invasive and more acceptable alternative to blood sampling for parents. Consequently, the GDG recommends the use of transcutaneous bilirubinometers after 24 h of age to mitigate issues associated with blood sample collection in community settings. In cases where transcutaneous bilirubinometers are unavailable, serum bilirubin levels should be monitored and documented. Furthermore, the NICE guideline on “Postnatal care” suggests monitoring and recording the intensity of jaundice in infants aged 24 h and older, alongside assessing the overall well- being of the infant concerning hydration and alertness (177). The GDG contends that any healthcare professional can assume the responsibility for monitoring and recording the infant's bilirubin levels. It should be noted that TcB measurements may not be reliable in infants undergoing phototherapy due to its skin-bleaching effect. Therefore, further investigations are warranted to evaluate the effectiveness of transcutaneous bilirubinometry in this specific population. Additionally, limited data exists regarding the use of transcutaneous bilirubinometers in newborns who have received phototherapy. While some studies have reported improved correlation between TSB and TcB values when measurements are taken from shaded body sites, further research is needed to assess the accuracy of this approach in infants undergoing phototherapy. Notably, Tan and Dong conducted a study exclusively on Asian infants who had undergone phototherapy, yielding inconsistent findings. Some authors have recommended conducting transcutaneous measurements on body areas protected from light exposure during phototherapy to ensure accuracy (178). Yoruk and colleagues explored the reliability of Bilicare measurements by covering a portion of the skin to prevent exposure to phototherapy. Their study encompassed 171 late preterm and term newborns with a gestational age of ≥ 35 weeks. Their findings indicated that TcB measurements obtained from unexposed skin areas can be safely utilized in patients undergoing phototherapy (179).

Despite studies assessing the accuracy of transcutaneous bilirubin (TcB) measurements in neonates undergoing phototherapy (PT), certain aspects require further investigation. For instance, a recent study by Yoruk et al. did not address the reliability of TcB measurements obtained from exposed skin areas in neonates undergoing PT (180). Lucanova conducted a prospective study involving 150 Caucasian term neonates, during which 255 TSB and TcB measurements were taken 2 h after PT discontinuation. Measurements were obtained from the forehead, sternum, abdomen, and covered lower abdomen. Their findings highlighted that phototherapy significantly interferes with the accuracy of TcB measurements, even when conducted on unexposed skin areas. As such, TSB assessment remains imperative when considering hyperbilirubinemia treatment in this context (181).

Table 3 documents recent related studies on nine different types of TcB devices since 2014, along with the paper title, journal, year of publication, name of the instrument, technique and site of measurement.

**Table 3:** Recent related studies on different TcB meters.

S. no	Title	Year	Journal	Number of neonates	Instrument	Technique	Site of measurement	Conclusion
1	Influence of assessment site on measuring Transcutaneous Bilirubin (174)	2014	Einstein (Sao Polo)	58 (Full term)	Bilicheck	Transcutaneous	Forehead and Sternum	High accuracy at sternum and low at forehead.
2	Diagnostic properties of a portable point-of-care method to measure Bilirubin and a transcutaneous Bilirubinometer (182)	2021	Neonatology	149	Bilistick v/s JM 105	Invasive	Hell prick	Bilistick underestimated TSB and TcB overestimated TSB
3	Evaluation of a new Transcutaneous bilirubinometers in newborn infants (183)	2022	Scientific Reports	141 (Full term)	JAISY v/s JM 105	Transcutaneous	Forehead and Sternum	Accuarte in low to moderate bilirubin levels
4	Evaluation of the Kejian KJ-8000 bilirubinometer in an Australian study (184)	2020	Journal of Pediatrics and Child Health	201	Kejian KJ-8000	Transcutaneous	–	Underestimated at high TSB, infants <32 weeks gestation had a poor correlation and Non Caucasian reported overestimation of TcB.
5	Development of a mobile phone camera based TcB for low resource settings (185)	2022	Biomedical Optics express	37	Mobile (Picture)	Transcutaneous	–	Holds potential but needs improvement and future work.
6	Clinical utility of Transcutaneous Bilirubinometer in very low birth weight infants (186)	2016	Journal of Perinatal medicine	100	N/A	Transcutaneous	–	Reliable in very low birth weight newborns in the absence of phototherapy.
7	Accuracy of the Bilicare Transcutaneous Bilirubin as predischarge screening tool in healthy term and late preterm neonates (187)	2020	European Association of Perinatal medicine	–	Bilicare	Transcutaneous	–	Overestimate TSB values, 12 mg/dl and underestimate at higher TSB level
8	Evaluation of a Point-of-care test for Bilirubin in Malawi (188)	2022	Pediatrics	375	BiliSpec	Invasive	Heel prick	Accurate and reproducible but needs more future work.
9	The accuracy of Transcutaneous Bilirubinometer to identify hyperbilirubinemia in jaundiced neonates (189)	2022	European Association of Perinatal medicine	–	JM-103	Transcutaneous	Sternum	Correlation affected due to gestational age and post-natal age of hour

**Figure 9:**
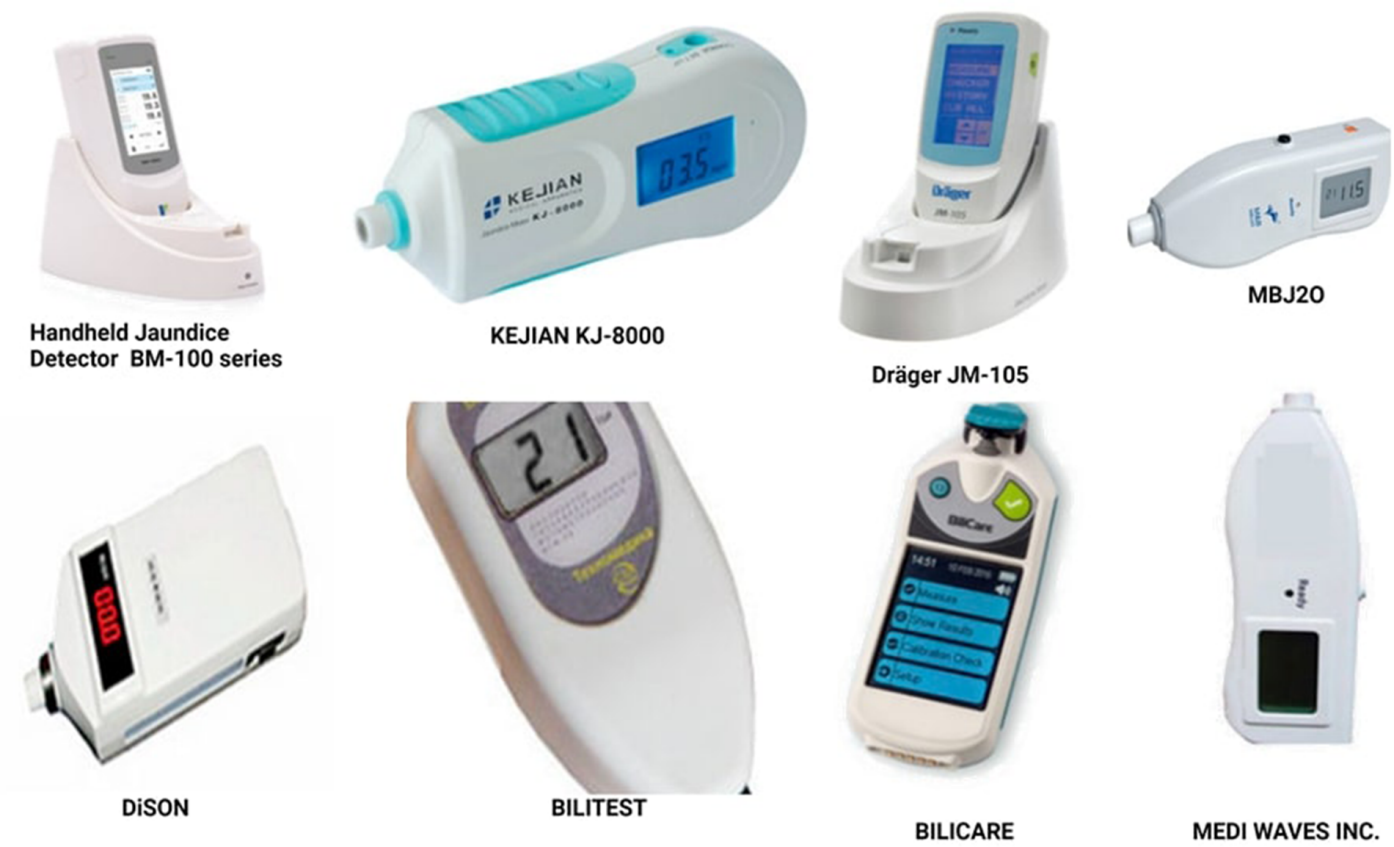
Prominent Key market players of transcutaneous bilirubinometers (192–199).

Costa-Posada et al. undertook a study to explore the utility of TcB in evaluating the effectiveness of PT on patched skin. They utilized a photo-opaque patch to cover a section of the skin (sternum) and performed simultaneous TcB and TSB measurements using the JM-105 bilirubin meter in 217 patients, nearly half of whom were preterm. Their investigation revealed that measuring TcB on patched skin facilitated the monitoring of PT response in both term and preterm infants. The patch, featuring a removable flap, enabled successive measurements without disturbing the patients (190). Raba et al. conducted a study to assess TcB measurement accuracy during and after PT in 196 preterm infants. While they identified strong correlations between TcB and TSB levels post-PT, during the PT phase, a considerable and clinically relevant disparity existed between the two measurement methods. Notably, this disparity improved significantly after PT cessation (191). Several observational studies have illustrated a robust correlation between Total Serum Bilirubin (TSB) and transcutaneous bilirubin (TcB) measurements. Furthermore, TcB measurements can be conducted by nurses or trained health workers with limited educational backgrounds, enhancing their accessibility and utility in clinical practice. Figure 9 provides an overview of key market players in the manufacturing of transcutaneous bilirubinometers.

A new invention in the field of TcB meters is that of “AJO-NEO” device, that collects the measurements from the neonatal nailbed. A prospective observational study was conducted over a 15-month duration in Kolkata, India, encompassing 2,092 neonates with gestational ages ranging from 28 to 40 weeks. The effective population size for this study was defined as 1,968 neonates (200). The primary objective of this research was to assess the efficacy of a novel non-invasive and non-contact bilirubin measurement device known as AJO-Neo as an alternative to the conventional invasive method involving biochemical estimation of TSB. This assessment was carried out in preterm and term neonates who exhibited hyperbilirubinemia due to various risk factors and/or were undergoing phototherapy. The study outcomes revealed a robust positive linear correlation between the bilirubin values obtained from AJO-Neo and those derived from TSB measurements and no statistically significant differences were observed when comparing measurements taken on the right and left nail beds (200). It is noteworthy that AJO-Neo exhibited substantial concordance with conventional TSB measurements, particularly when assessments were conducted during phototherapy.

*Limitations:*

1. Skin Pigmentation and Race: Numerous studies have investigated the correlation between TcB and TSB measurements in diverse populations, employing devices such as BiliChek and JM-103. However, it is noteworthy that the JM-103 device tends to overestimate TSB levels in infants with darker skin pigmentation. While TcB measurements are commonly used as an initial screening tool, this practice may result in an increased number of unnecessary TSB measurements. A study by Wainer et al. (132) explored the impact of skin tone on JM-103 performance, revealing that infants with medium skin tones exhibited the highest precision and least bias. Conversely, the lighter skin tone group tended to underestimate TSB levels, while the darker skin tone group tended to overestimate them.
2. Preterm and Low Birth Weight Infants (Gestational age, Malnutrition, etc.): The reliability of TcB measurements in infants with birth weights under 1,000 g or gestational ages of 28 weeks has been a subject of investigation. While some studies have suggested reduced reliability in these cases, others have not supported this finding (186, 201). Several studies have demonstrated the effectiveness of TcB measurements in both low birth weight (LBW) and extremely low birth weight (ELBW) infants within neonatal intensive care units (NICUs) (111, 200). By using appropriate cutoff values, TcB measurements accurately identified infants requiring TSB measurements or phototherapy.
3. Ambient Light: Ambient light, especially direct sunlight or strong artificial light, can affect the accuracy of TcB measurements, particularly when taken from the forehead. The presence of bright light can interfere with the measurements, leading to potential inaccuracies. In a study that aimed to investigate whether natural daylight exposure had an impact on the precision and consistency of TcB measurements taken at both the forehead and sternum regions. A cohort of 107 full-term newborn infants was divided into two distinct groups for analysis. Group I (*N* = 59) consisted of infants placed near a window, occasionally exposed to direct daylight, while Group II (*N* = 48) included infants positioned on the door side of the room, devoid of direct daylight exposure, all within the initial week of life (202). The findings of this study highlighted the significance of daylight as a substantial influencing factor on TcB measurements obtained at the forehead. This factor was found to contribute to a suboptimal correlation between TcB readings at the forehead and serum bilirubin concentrations. Consequently, we advocate for the necessity of acquiring TcB readings at both the forehead and sternum, at a minimum, as a means to enhance the precision and dependability of such measurements in neonates (202). To mitigate the impact of ambient light on TcB measurements, it is essential to control the lighting conditions during the measurement process. Some TcB devices come equipped with shields or covers to block out ambient light, ensuring more accurate readings. Additionally, conducting TcB measurements in a dimly lit or controlled environment can help minimize the influence of ambient light on the results. Researchers and healthcare professionals should be aware of the potential impact of ambient light and take measures to standardize the measurement conditions to obtain reliable TcB readings.
4. Effect of TSB Level: TcB measurements tend to be less accurate and may underestimate TSB levels at higher concentrations (203). Consequently, as TSB levels increase, the rate of false-negative TcB results may rise. Nevertheless, when employing suitable cutoff values, TcB measurements remain effective as a screening tool, even when TSB levels exceed 15 mg/dl.
5. Effect of Phototherapy on TcB Measurements: The use of phototherapy can lighten an infant's skin, potentially compromising the reliability of visual assessments of jaundice and TcB measurements (181, 203). However, if specific areas of the skin remain shielded during phototherapy, TcB measurements from those areas can be employed to monitor treatment response. In a prospective observational study, the suitability of TCB measurement was assessed as a tool for evaluating the effectiveness of phototherapy on a localized skin patch (198). Specifically, a photo-opaque patch was applied to a portion of the infant's skin (sternum), and multiple simultaneous measurements of TCB and TSB were conducted using the JM-105 bilirubinometer. This method, referred to as “patched skin transcutaneous bilirubin” (PTCB) measurement, proves valuable for monitoring the response to phototherapy in both term and preterm infants. Importantly, the use of a patch with a removable flap facilitates successive measurements without causing discomfort or disturbance to the patients (198). A systematic review of studies was done in order to compare the TcB devices with total serum bilirubin TSB measurements in infants undergoing phototherapy or in the post-phototherapy phase. The included studies involved infants with a gestational age of ≥34 weeks. The findings of which indicate that TcB devices exhibit reduced accuracy in estimating serum bilirubin levels in infants undergoing phototherapy compared to their documented accuracy in the pre-phototherapy period (204).
6. Site of Sampling: Both BiliChek and JM-103 devices recommend obtaining TcB measurements from either the forehead or sternum (175). In a study involving 475 infants comparing JM-103 measurements taken from the forehead and sternum with TSB measurements, the Pearson correlation coefficients were higher for measurements from the sternum (0.953) than for those from the forehead (0.914) (123). Given that the forehead is exposed to ambient light both in the nursery and post- discharge, while the sternum is typically covered, measurements from the sternum are likely the preferable choice. Another study involving 31 infants comparing outpatient BiliChek measurements from the brow and sternum revealed that brow readings were approximately 20% lower than TSB values, whereas chest readings were only around 5% lower.

### 4.4. Digital imaging and mobile applications

**Figure 10:**
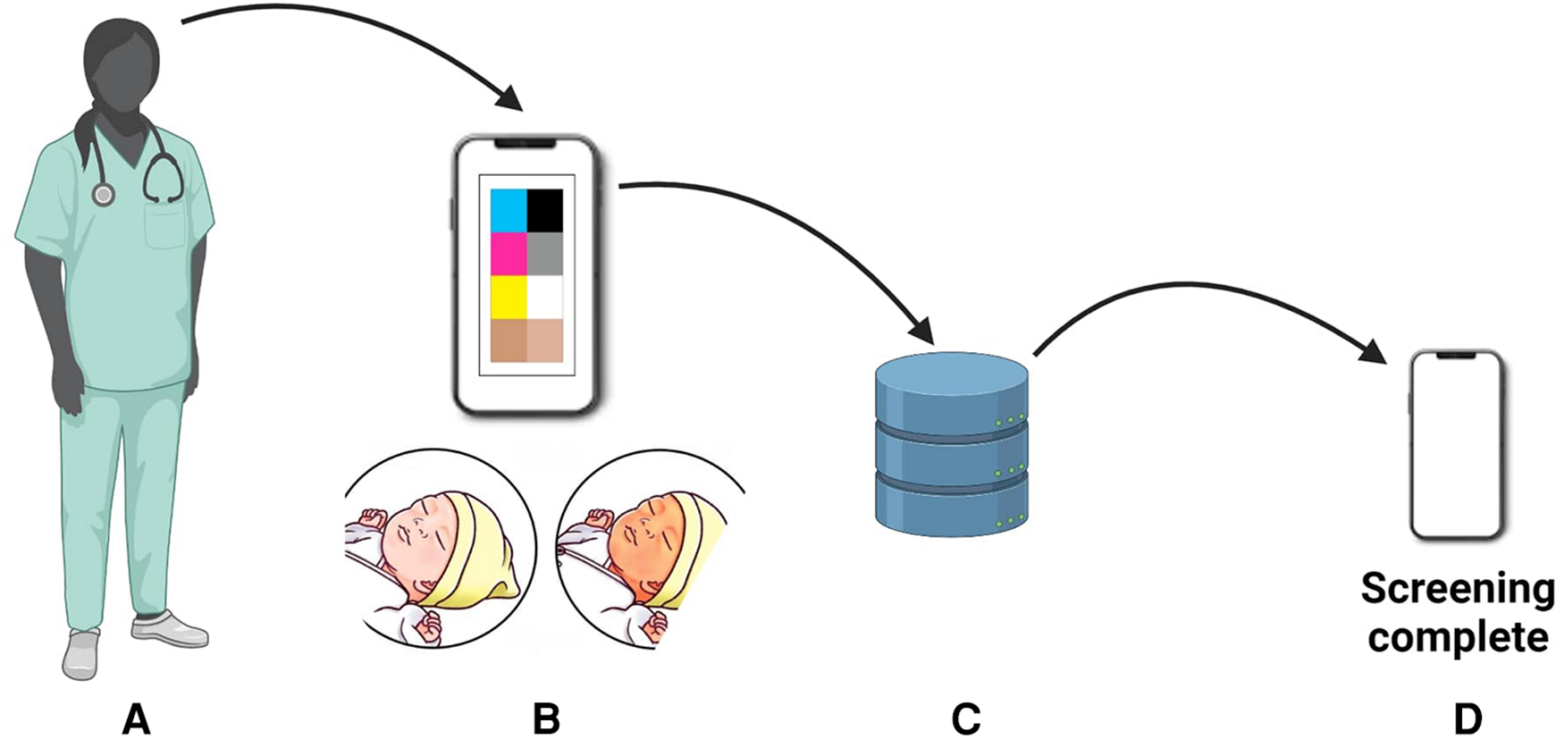
Workflow on bilirubin estimation: (**A**) newborn photography, (**B**) color calibration, (**C**) database comparison, and (**D**) bilirubin estimation for neonatal jaundice assessment.

Digital imaging represents a non-invasive approach to hyperbilirubinemia screening (205). It involves capturing images and analyzing them using specialized applications to assess color and identify jaundice. One such is the optical imaging of the conjunctiva for bilirubin analysis that represents an alternative to the transcutaneous bilirubinometer BiliChek (205). A prospective cross-sectional study by Aune et al. involved 302 newborns, including 76 with severe jaundice (206). The researchers developed a smartphone-based tool named Picterus, which employed digital images to estimate bilirubin levels and detect severe jaundice with high sensitivity. This innovative approach showcased promising results for neonatal jaundice screening in Caucasian newborns. The bilirubin estimates derived from the images exhibited a strong correlation with TSB levels. Picterus demonstrated a remarkable sensitivity of 100% in identifying participants with severe jaundice (defined as TSB exceeding 250 µmol/L) and a specificity of 69% (206). For a detailed workflow of this system, refer to Figure 10.

**Figure 11:**
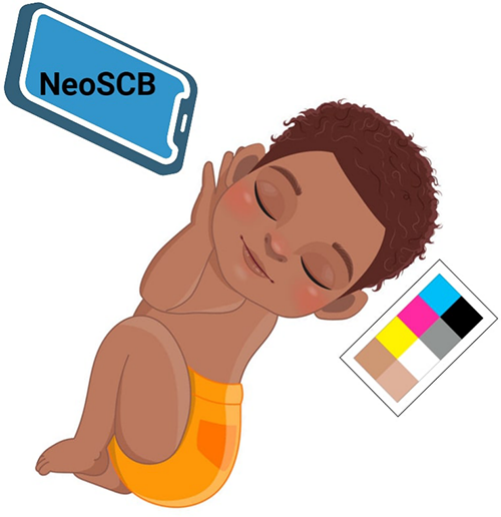
Enhancing neonatal jaundice screening while using NeoSCB App to investigate ambient lighting and color factors.

In a collaborative clinical pilot, researchers conducted comprehensive studies on neonatal jaundice screening utilizing smartphone technology. The pilot involved 37 newborns at University College Hospital London, and a year-long study was carried out on 724 newborns in Ghana (207). To capture precise eye images, the research team employed a Samsung Galaxy SB smartphone, capturing two images per infant's eye—one with the LED flash activated and one without, effectively mitigating ambient light influence through ambient subtraction techniques. The diagnostic accuracy of the Skin Color Bilirubin (SCB) level measured by the app was validated against a Dräger JM-105 jaundice meter and the gold standard laboratory blood test, which determines TSB levels (Figure 11). During the study, the researchers fine-tuned the neoSCB app, optimizing the subtracted signal-to-noise ratio (SSNR) for real-time quality control. Additionally, they introduced a feature enabling users to zoom in on captured images and manually select an area of interest on the sclera to obtain a real-time calculated SCB value. The neoSCB app's diagnostic algorithm underwent validation, and further enhancements to the user interface are underway to enhance its usability for healthcare practitioners. The researchers envision the app's potential for independent use or integration into established maternal-child healthcare applications to augment their functionality (208). Yan et al. conducted a research investigation aimed at assessing the impact of a smartphone-based neonatal jaundice screening program conducted outside of the hospital environment (209). Their study involved a cohort comprising 1,424 mother-infant dyads, encompassing 1,424 mothers and 1,424 newborns. The outcomes of the study revealed that the implementation of the smartphone-based out-of-hospital screening approach for neonatal hyperbilirubinemia was associated with a decrease in neonatal readmission rates within the 30-day period following the initial discharge. Furthermore, it provided some degree of improvement in maternal mental health. Notably, this information has been previously published and cited accordingly (209). In conclusion, digital imaging techniques offer a promising avenue for non-invasive, real-time, and accurate neonatal jaundice screening. These innovations are rapidly becoming indispensable tools for neonatologists and pediatricians in effectively managing this condition.

One of the studies, aimed to assess the potential utility of a smartphone's embedded camera as a screening tool for neonatal hyperbilirubinemia. A total of 64 randomly selected healthy Caucasian newborns meeting specific gestational and age criteria, with parental consent obtained, were included in the research (210). Images of the glabella were captured using an iPhone 6 under three conditions: direct pressure, dermatoscope, and dermatoscope with a Wratten No. 11 filter. The color intensities of the red, green, and blue channels in each image were then compared to bilirubin levels. The findings revealed that only the intensities of the green and blue channels acquired with the dermatoscope showed a significant correlation with bilirubin measurements (*p < *0*.*001), with respective Pearson's correlation coefficients of 0.59 and 0.48. Discrimination limits of 212 and 190 for the green and blue channels, respectively, demonstrated high sensitivity (100% for green and 90.9% for blue) but relatively lower specificity (62.5% for green and 60% for blue) for detecting plasma bilirubin levels above 205 µmol/L (210). In conclusion, the study suggested that a smartphone equipped with a dermatoscope, ensuring consistent light conditions, may serve as a straightforward screening tool for neonatal hyperbilirubinemia. However, further refinement is necessary before its clinical application can be considered.

In another cross-sectional study encompassing 100 neonates, patient data was collected and where the average gestational age of the neonates in the cohort was 39 weeks (205). Total bilirubin levels were assessed utilizing a transcutaneous bilirubinometer, which entailed measurements on the forehead and optical imaging scans of the conjunctiva of the eyes, denoted as BiliChek and BiliCapture, respectively. Subsequently, blood samples were procured from these neonatal subjects and subjected to laboratory analysis to ascertain the levels of TSB. The concentration of bilirubin, as determined from serum samples, BiliChek, and BiliCapture, were recorded and robust correlations were observed between TSB and both BiliChek and BiliCapture. Evaluation through Bland-Altman plots demonstrated a high level of concordance when comparing bilirubin values obtained from both BiliChek and BiliCapture devices. Furthermore, bilirubin measurement was subjected to sensitivity and specificity analyses, yielding values of 88% and 76% for BiliChek and 92% and 75.6% for BiliCapture, respectively. These findings highlight the safety and viability of optical imaging of the conjunctiva as an alternative to conventional laboratory- based bilirubin assays and the transcutaneous bilirubinometer BiliChek (205). Mobile applications have revolutionized neonatal jaundice diagnosis by harnessing the capabilities of smartphone and tablet cameras. These applications empower parents and healthcare professionals to monitor jaundice conveniently at home or in hospital settings. Among the notable applications is the Bilicam app, developed by researchers at the University of Washington. Leveraging the smartphone's camera and flash, Bilicam captures skin images and employs an algorithm to estimate bilirubin levels. Another noteworthy app, BiliScreen, utilizes a smartphone camera and a specialized filter to capture eye images for estimating blood bilirubin levels. Clinical trials have demonstrated promising results for this innovation, also developed by University of Washington researchers. While mobile applications offer numerous advantages over traditional methods—such as blood tests and visual inspection—such as non-invasiveness, user-friendliness, and real-time feedback, they are not without limitations. These apps depend on precise color calibration and specific lighting conditions, and results may vary based on skin tone and other factors. Nevertheless, their potential to reduce hospital readmissions and healthcare costs underscores their importance in neonatal jaundice management.

*Limitations:* The principal objective of these device applications is to facilitate timely and frequent screening. The Mobile App Predicting Bilirubin (MAPB) measurement, positioned as a screening tool, possesses characteristics that render it non-invasive, devoid of physical contact, and amenable to user interaction. Nevertheless, it is not devoid of constraints. This approach necessitates the installation of a dedicated application on a smartphone, thus mandating the prerequisite possession of a smartphone within the family unit. Furthermore, it demands a certain degree of technological literacy. On the contrary, emerging Icterometers, exemplified by the Bili-strip and Bili-ruler, circumvent the necessity of smartphone reliance, emerging as cost-effective instruments in LMIC's (90, 142). However, it is imperative to subject them to further scrutiny encompassing diverse ethnicities and variations in skin pigmentation. Advanced technological modalities, such as wearable devices designed for continuous monitoring, are under evaluation but are poised to cater primarily to high-income households (211). Pertinent variables that may influence MAPB measurements encompass skin pigmentation, the specific device employed, and the ambient lighting conditions. Among the studies incorporated in this review, one study reported the effectiveness of MAPB measurements in predicting TSB levels in neonates of varying ethnic backgrounds (212). The study revealed that although correlations exhibited similarities across distinct ethnic groups, a marginal augmentation was observed in white neonates (142). A separate investigation noted that correlations in the Caucasian subgroup surpassed those in the non-Caucasian group (206) However, it is noteworthy that the latter study encompassed a non-Caucasian infant cohort comprising merely 23% of the total sample size.

Certain researchers have explored the utilization of scleral imaging, positing certain advantages over cutaneous assessments due to the absence of melanin or hemoglobin chromophores. The incorrect estimation of TSB values, stemming from melanin concentration in the skin, emerges as a critical concern necessitating future attention. It is imperative to incorporate melanin levels in the skin as a pivotal component of mathematical models in all forthcoming iterations of MAPB devices. The spectrum of included studies manifests a global footprint, with a majority demonstrating concordant correlation coefficients. Notably, studies originating from India reported comparatively lower correlation coefficients (137, 205, 213–218). The role of ambient lighting assumes significance, as the procedure entails capturing digital images. A subset of the included studies reported more favorable correlation coefficients during daylight hours compared to night (216). The employment of a color calibration card serves to mitigate variations in ambient lighting conditions, thereby facilitating consistent image capture. Researchers have also introduced innovative models aimed at ameliorating the influence of ambient light (214). Several of the included studies conducted comparisons between the accuracy of Transcutaneous Bilirubin (TcB) and MAPB in their respective study cohorts. These investigations consistently favored TcB over MAPB in the estimation of TSB values (205–207). The reported correlations between TcB and TSB exhibited a range from 0.77 to 0.97, with the highest concordance observed in neonates during their initial hospitalization and the lowest in outpatient settings, where MAPB measurements are anticipated for employment (122, 130, 161, 219–220).

## 5. Open problems for research on transcutaneous bilirubinometers

In light of the foregoing discussion regarding limited access to adequate healthcare infrastructure, healthcare professionals, and various societal and economic barriers, a range of open research challenges emerges with respect to the broader applicability and acceptance of transcutaneous bilirubinometers (TcB) in clinical practice. The following elucidates these open problems:

### 5.1. Improving the clinical utility of transcutaneous bilirubinometers

Numerous investigations have delved into the clinical utility of TcB as a non-invasive alternative to invasive blood sampling for bilirubin level measurement. Notable studies by Shabuj et al. and Wong et al. have demonstrated the accuracy of TcB for screening neonatal hyperbilirubinemia in preterm infants, albeit acknowledging its limitations in replacing invasive blood sampling for diagnostic purposes (135, 221). These findings underline the need for innovative approaches to augment the clinical effectiveness of TcB beyond its current role as a screening tool. Research endeavors should explore avenues for harnessing this technology to furnish precise and reliable measurements capable of informing clinical decision-making, and potentially supplanting invasive blood sampling in select scenarios.

### 5.2. Addressing limitations in neonatal screening during and after phototherapy

A conspicuous limitation of TcB technology is its diminished effectiveness in screening neonates undergoing or post phototherapy. There exists a pressing need for research aimed at enhancing the accuracy and reliability of TcB devices in the presence of phototherapy (222). This necessitates an investigation into the challenges confronting the accuracy of TcB during phototherapy and the formulation of strategies to surmount these constraints. Moreover, the exploration of alternative techniques or modifications to existing technology capable of furnishing precise and reliable bilirubin measurements during phototherapy is imperative (223). A comprehensive understanding of the photo breakdown of bilirubin concerning TcB measurements and the development of techniques to account for this phenomenon would significantly enhance the clinical applicability of TcB devices.

### 5.3. Understanding the impact of phototherapy on the relation between TcB and TSB

The relationship between TcB and TSB is subject to various influences, including the degradation of bilirubin during phototherapy. Extensive research efforts are warranted to elucidate this relationship and develop methodologies that enable the accurate correlation of TcB measurements with TSB levels, particularly in the context of phototherapy (204). Consequently, it is essential to acquire insights into the factors exerting an influence on this relationship and devise techniques to precisely estimate TSB levels based on TcB readings in the presence of phototherapy.

### 5.4. Available technology works on selective neonatal population in terms of gestational age at birth and ethnicity/race

Current limitations in transcutaneous bilirubinometer technology pose challenges in its adaptability to diverse neonatal populations, characterized by variations in gestational age at birth and ethnicity/race, as indicated by several sources (224–227). To address this issue, research endeavors should prioritize the development and validation of transcutaneous bilirubinometers capable of furnishing accurate measurements across a broad spectrum of gestational ages and diverse ethnic/racial backgrounds (37, 228). Such endeavors aim to ensure impartial access to dependable bilirubin screening for neonates, irrespective of their demographic attributes. Although the influence of gestational age at birth on bilirubin metabolism in neonates is well-acknowledged, the specific impact of gestational age on transcutaneous bilirubin measurements remains incompletely understood (229). Further research is imperative to explore the intricate relationship between gestational age and transcutaneous bilirubin measurements, encompassing the identification of potential confounding variables. Such investigations pave the way for the formulation of gestational age-specific calibration algorithms, thereby refining the accuracy of transcutaneous bilirubinometers across distinct gestational age cohorts. While ethnicity and race have been implicated in bilirubin metabolism, potentially affecting the precision of transcutaneous bilirubin measurements, a more comprehensive research approach is warranted to scrutinize the ramifications of ethnicity/race on transcutaneous bilirubin readings. Additionally, this research should endeavor to identify requisite adjustments or calibration methods tailored to different ethnic or racial groups, thereby reinforcing the reliability and precision of transcutaneous bilirubinometers across diverse population strata. The establishment of precise reference ranges assumes pivotal importance in the clinical interpretation of transcutaneous bilirubin measurements. Presently, reference ranges may inadequately encapsulate the variabilities observed in disparate neonatal populations. Research initiatives should refocus their efforts on the formulation of population-specific reference ranges, taking into careful consideration factors such as gestational age at birth, ethnicity/race, and other pertinent variables. By doing so, clinicians would be empowered to make more judicious decisions grounded in transcutaneous bilirubin measurements tailored to specific population subsets. Genetic factors hold the potential to introduce inter-individual variances in bilirubin metabolism and skin properties, thereby potentially influencing the precision of transcutaneous bilirubin measurements. A meticulous exploration of the genetic determinants associated with transcutaneous bilirubinometry is warranted to uncover genetic markers or variants with a discernible impact on bilirubin measurements. These revelations can subsequently inform the development of personalized approaches for calibration or interpretation, thereby advancing our comprehension of the genetic underpinnings of transcutaneous bilirubinometry and affording avenues for individualized neonatal care.

### 5.5. Development of continuous monitoring of bilirubin

Presently, a noticeable absence exists in the realm of continuous monitoring systems designed to assess bilirubin levels—a deficiency that holds potential for enhancing the management of neonatal hyperbilirubinemia. In a notable study by Inamori et al., a wearable transcutaneous bilirubinometer endowed with the added functionalities of oxygen saturation (SpO2) and heart rate (HR) sensing was introduced, demonstrating its capability to measure bilirubin levels during phototherapy (211). To address this gap, research endeavors should be channeled towards the development of innovative technologies or methodologies that enable non-invasive, continuous monitoring of bilirubin levels. Moreover, it is imperative to delve into the feasibility of real-time, non-invasive monitoring systems that can furnish clinicians with continuous data regarding bilirubin levels, thereby affording a more accurate and timely basis for interventions.

### 5.6. Understanding the spatio-temporal heterogeneity of TcB results

Transcutaneous bilirubin measurements, governed by a multitude of factors, engender intricate spatio- temporal heterogeneity in their outcomes. A comprehensive identification and characterization of these factors are imperative, encompassing variables such as gestational age at birth, ethnicity/race, and other pertinent determinants, with the overarching goal of enhancing the accuracy and reliability of TcB measurements. This endeavor necessitates the development of algorithms or models capable of accommodating these factors, consequently augmenting the clinical utility of transcutaneous bilirubinometers.

### 5.7. Improving access and applicability in low-resource settings

The present landscape of TcB technology raises concerns regarding its adaptability for extensive deployment in low-resource settings. To redress this issue, research should pivot towards the creation of cost-effective, robust, and user-friendly devices, finely attuned to the unique requisites of low-resource settings. This holistic approach includes tackling impediments like power source accessibility, device maintenance, and adaptability to diverse healthcare infrastructures. Bridging this technological divide stands to fortify accessibility to neonatal bilirubin screening in resource-constrained regions. They have potential limitations associated with the accuracy and precision of TcB, implying that it may not be inherently suited for application in low-resource settings marked by constrained access to laboratory testing. Consequently, the suitability of TcB in low-resource settings has been cast into doubt, prompting contemplation of alternative strategies such as clinical assessment. Addressing these shortcomings in available technology tailored to low-resource settings, particularly primary healthcare centers, remains an incisive research imperative. Further inquiry is warranted to engineer cost-effective, portable technologies capable of delivering accurate bilirubin level measurements within the constraints of resource-limited settings.

### 5.8. Development of at-home-based screening using transcutaneous bilirubinometers

Another pivotal avenue of research revolves around the development of at-home-based methods for transcutaneous bilirubin screening. Such innovation has the potential to empower parents and caregivers, enabling them to conveniently and regularly monitor their infants’ bilirubin levels, potentially mitigating the need for hospital visits and invasive blood sampling procedures. A 2020 study expounded on the challenges associated with implementing home-based neonatal jaundice screening in resource-scarce settings, underscoring the demand for cost-effective and user-friendly screening tools deployable by community health workers (230). In a separate study conducted in 2022 within rural India, the feasibility and efficacy of a home-based screening program for neonatal jaundice were validated, yielding tangible reductions in the incidence of severe jaundice and associated hospitalizations (231). While a plethora of research endeavors focuses on the development of at-home screening tools for neonatal jaundice, the market has yet to yield a definitive and universally trusted solution for home-based screening. Consequently, a pressing research mandate revolves around the exploration of the feasibility, accuracy, and user acceptance of at-home-based transcutaneous bilirubinometers, with the ultimate objective of markedly enhancing the accessibility and convenience of neonatal bilirubin screening.

## 6. Challenges and future perspectives

### 6.1. Economic and social barriers

Economic constraints represent a substantial impediment to the screening and diagnosis of jaundice, particularly in low- and middle-income countries. The costs associated with screening and diagnostic tests can prove exorbitant, rendering them financially inaccessible for many families. In such circumstances, families are often faced with the agonizing choice of allocating limited resources to these tests or addressing basic necessities such as sustenance and shelter. Additionally, healthcare systems may necessitate increased financial allocation to facilitate widespread access to these tests, a particularly challenging feat in remote or rural regions. Treatment costs present another formidable economic barrier. Severe jaundice cases may mandate hospitalization and phototherapy, incurring substantial expenses. In regions where health insurance coverage is either scarce or non-existent, families may find themselves unable to bear the treatment expenses, thereby culminating in delayed intervention and the potential onset of severe complications.

Social barriers, too, exert a notable influence on jaundice screening and diagnosis. One salient social impediment is the lack of awareness regarding the significance of screening and treatment for jaundice. Certain communities harbor misconceptions concerning the etiology and therapeutic measures associated with jaundice. Consequently, families may either eschew medical attention for their newborns or defer seeking care until the ailment reaches a critical stage. Language and cultural disparities can further compound the challenges associated with jaundice screening and diagnosis, complicating the provision of apt healthcare. Moreover, cultural beliefs may impede families' willingness to seek medical care or adhere to treatment recommendations.

In summation, economic and social barriers cast a shadow over the screening and diagnosis of jaundice, particularly in low- and middle-income nations. Overcoming these barriers mandates a concerted healthcare system commitment to rendering affordable and accessible screening, diagnostic tests, and treatment for jaundice. Concurrently, education and awareness campaigns assume pivotal roles in surmounting social and cultural impediments, engendering an understanding of the imperative of early jaundice screening and treatment.

### 6.2. Technological barriers

Several technological hurdles encumber the precise screening and diagnosis of jaundice. An inherent impediment lies in the absence of standardized screening protocols. While jaundice screening is universally recommended for all neonates, the absence of uniform screening methodologies presents a challenge. Variability prevails, with some hospitals relying on visual inspection of the skin, while others employ TcB devices. This dearth of standardization complicates result comparisons across different healthcare facilities and regions, potentially fostering screening, diagnostic and treatment inconsistencies. A further technological obstacle manifests in the limited accessibility of screening devices, particularly in resource- scarce settings. Although TCB and TSB are efficacious in jaundice diagnosis, their cost, requirement for specialized equipment, and dependence on trained personnel render them inaccessible in low-resource settings. Consequently, healthcare providers in such settings may have to solely rely on visual inspection, which may yield less accurate results. Phototherapy, a standard jaundice treatment involving the use of specialized equipment and light exposure to break down bilirubin, presents its own set of challenges. One of the prominent challenges encountered pertains to the limited accessibility of phototherapy apparatus and adequately trained personnel within LMIC's. This situation underscores the inherent predicament that, even in cases where screening procedures prove effective, the absence of essential therapeutic equipment impedes the ability to carry out the complete treatment process. The screening of patients, however, remains only a partial facet of patient management when the capacity for effective treatment is lacking. It necessitates vigilant monitoring to ensure precise light dosages. Furthermore, some infants may exhibit inadequate responses to phototherapy, necessitating more invasive and perilous treatments such as exchange transfusion.

In conclusion, technological impediments loom large in the accurate screening and diagnosis of neonatal jaundice. Redressing these impediments necessitates a comprehensive effort towards standardizing screening protocols, enhancing the widespread availability of screening devices, and innovating novel treatments for severe jaundice cases. By undertaking these measures, we can ensure that every neonate receives the highest caliber of care and treatment.

## 7. Summary

Non-invasive bilirubin screening has been a subject of exploration for the past three decades, primarily deployed within well-endowed tertiary care centers. However, it remains limited, both on a national and international scale, largely constrained by technical deficiencies and financial constraints. Consequently, their presence in primary and secondary healthcare sectors remains sporadic. The technical limitations encompass the challenge of providing reliable measurements in premature infants and individuals of non-Caucasian descent, primarily stemming from variations in skin color, density, and thickness. Further complexities arise due to the inherent variability associated with modalities and specific treatments, resulting in intricate spatial and temporal fluctuations in bilirubin measurements, particularly evident before, during, and after therapeutic interventions. The calibration, interpretation, and harmonization of these devices pose additional challenges. The overarching factors of cost, ease of use, and the ability to replicate results further contribute to their constrained adoption. Furthermore, the scarcity of phototherapy equipment and qualified healthcare personnels for treatment underscores the inherent limitations of screening, as screening alone holds limited clinical value in the absence of adequate resources for intervention. Research endeavors in bilirubin determination methods continue to evolve, with a surge in publications over recent years, all aimed at devising novel and enhanced strategies. From a commercial perspective, the recommendation surfaces for multiplex bilirubin determination devices capable of encompassing other clinically significant analytes. While research in the realm of non-invasive technologies continues to advance, it is essential to acknowledge that this approach primarily serves as a screening method, while TSB maintains its position as the gold standard for diagnostic purposes. Non-invasive methods can contribute to the screening process; however, they should not be exclusively depended upon. TSB remains indispensable as a confirmatory test, underscoring its role in clinical diagnosis. Therefore, non-invasive techniques should be considered as auxiliary clinical tools to facilitate assessment rather than standalone methods for treatment. Looking ahead, future directions in TcB research necessitate a focus on refining device accuracy by accounting for variables such as skin pigmentation, gestational age, and postnatal age. Exploratory avenues also extend to the development of innovative technologies, including but not limited to multispectral imaging and fluorescence spectroscopy, as potential means for bilirubin estimation in neonatal populations. Additionally, there is an evident and urgent requirement for the execution of a more extensive range of studies to evaluate the clinical efficacy of transcutaneous bilirubinometers within various contexts, populations, assessment site methodologies, and device designs.
